# Surface Functionalization of ITO for Dual-Mode Hypoxia-Associated Cancer Biomarker Detection

**DOI:** 10.3390/bios15030186

**Published:** 2025-03-14

**Authors:** Edmunds Zutis, Gunita Paidere, Rihards Ruska, Toms Freimanis, Janis Cipa, Raivis Zalubovskis, Maira Elksne, Kaspars Tars, Andris Kazaks, Janis Leitans, Anatolijs Sarakovskis, Andris Anspoks

**Affiliations:** 1 Institute of Solid State Physics, University of Latvia, 8 Kengaraga Street, LV-1063 Riga, Latvia; 2 Latvian Institute of Organic Synthesis, 21 Aizkraukles Street, LV-1006 Riga, Latvia; 3 Institute of Chemistry and Chemical Technology, Faculty of Natural Sciences and Technology, Riga Technical University, P. Valdena iela 3, LV-1048 Riga, Latvia; 4 Latvian Biomedical Research and Study Centre, 1 k-1 Ratsupites Street, LV-1067 Riga, Latvia

**Keywords:** indium tin oxide, carbonic anhydrase IX, sandwich immunoassay, chemiluminescence, electrochemistry

## Abstract

Indium tin oxide (ITO) is a transparent conducting material with exceptional electrical and optical properties, widely used in biosensing and bioelectronics. Functionalization of ITO with linker molecules enables covalent attachment of biomolecules, allowing for dual-mode optical and electrochemical detection. Carbonic anhydrase IX (CA IX), a transmembrane enzyme overexpressed in hypoxic tumors, is a promising biomarker for cancer diagnostics due to its restricted expression in normal tissues. However, conventional detection methods are time-intensive and unsuitable for point-of-care applications. In this study, ITO surfaces were functionalized using silane-based chemistry to immobilize CA IX-specific antibodies, creating a novel biosensing platform. The biosensor utilized a secondary horseradish peroxidase (HRP)-conjugated antibody to catalyze the oxidation of luminol in the presence of hydrogen peroxide, producing a chemiluminescent and electrochemical signal. Characterization of the biosensor via a dual-mode optical and electrochemical approach revealed efficient antibody immobilization. Due to the high variation observed in the optical approach, limit of detection (LOD) experiments were conducted exclusively with electrochemistry, yielding an LOD of 266.4 ng/mL. These findings demonstrate the potential of ITO-based electrochemical biosensors for sensitive and selective CA IX detection, highlighting their applicability in cancer diagnostics and other biomedical fields.

## 1. Introduction

Indium tin oxide (ITO) is a widely used transparent conducting material with applications in various fields, including biosensing and bioelectronics [1]. Its high electrical conductivity, excellent optical transparency, stable electrochemical and physical properties, and strong substrate adhesion make it a promising candidate for both electrochemical and optical sensing applications [2]. While ITO holds significant potential for biosensing applications, its surface requires additional modification steps to ensure the attachment of detection molecules, thereby providing high specificity for analysis [3]. However, most current functionalization strategies for ITO, such as electrophoretic and electrochemical deposition, involve the application of an additional conductive layer on the ITO surface to ensure covalent bonding of biorecognition elements, increasing fabrication costs [4].

Self-assembled monolayers (SAMs) provide a promising strategy for the functionalization of ITO surfaces, enabling the covalent attachment of biorecognition elements [5]. Among organic SAMs, silane-based molecules have demonstrated good reproducibility, stability, and ease of preparation. This method is based on ITO surface functionalization with hydroxyl groups through exposure to an oxidation-inducing environment, after which a silane-based reagent can cross-link with the surface to form Si-O bonds [6]. The terminal group of the silane SAMs is often used to link biorecognition elements such as antibodies, proteins, or aptamers [7].

Carbonic anhydrase IX (CA IX) is a transmembrane enzyme overexpressed in various tumor types, including renal cell carcinoma, malignant melanoma, non-small cell lung cancer, and breast cancer. CA IX plays a crucial role in regulating tumor pH and is strongly associated with hypoxic tumor microenvironments [8,9]. Moreover, high CA IX expression is generally correlated with poor prognosis and a shorter disease-free interval following therapy. While CA IX is also expressed in normal tissue, its expression is limited and primarily restricted to the gastrointestinal tract [10,11]. This differential expression makes CA IX a valuable biomarker for cancer diagnosis and prognosis.

Although CA IX’s role as a membrane-bound biomarker for hypoxia-related cancers is well established, its diagnostic potential as a circulating biomarker in bodily fluids, for instance, blood and urine, has been less explored. Free CA IX, presumably released into body fluids due to the proteolytic cleavage of its extracellular domain, has been detected in blood and urine, with significantly higher levels observed in cancer patients than in healthy individuals [12,13]. This suggests that quantifying free CA IX could serve as a minimally invasive or non-invasive platform for tumor monitoring, particularly for detecting residual tumor cells after surgery or chemotherapy.

However, the detection of free CA IX presents technical challenges due to its low circulating levels [12]. Currently, methods such as immunoprecipitation and enzyme-linked immunosorbent assays (ELISAs) are commonly employed for CA IX measurements [12,14]. While ELISA offers high specificity and sensitivity, it requires multiple incubation steps, specialized laboratory infrastructure, and trained personnel, making it impractical for point-of-care diagnostics. Immunoprecipitation methods, on the other hand, can suffer from high sample preparation times and variability [15].

Electrochemical biosensors offer a promising alternative due to their high sensitivity, specificity, low cost, and potential for miniaturization [16]. However, electrochemical methods can be susceptible to signal interference and limited selectivity in complex biological samples, which may impact their performance in real-world scenarios [17]. Combining electrochemical detection with optical methods in a single biosensor has emerged as a powerful tool to overcome these limitations, enhancing both the detection robustness and diagnostic accuracy [18]. Recent studies have shown that this dual-modality approach enhances sensitivity and specificity and extends the detection range by integrating complementary detection principles [19]. Additionally, cross-validation between the two methods significantly improves accuracy, while the inclusion of optical detection reduces false positive signals, enhancing the overall specificity and reliability of the biosensor [20]. This synergy has proven particularly effective in biomarker detection, especially in cancer diagnostics. Notably, it has been applied for the detection of circulating tumor DNA [21], epidermal growth factor receptor 2 breast biomarker [22] and colon cancer inhibitor genes [23], demonstrating high detection reliability and strong resistance to false results. However, to date, no dual-mode biosensors for the detection of CA IX have been reported in the literature.

In this study, we introduce a simplified, silane-based functionalization strategy for ITO, enabling the direct covalent immobilization of CA IX-specific capture antibodies. A sandwich immunoassay approach is employed, where a secondary horseradish peroxidase (HRP)-conjugated antibody catalyzes the oxidation of luminol in the presence of hydrogen peroxide, producing both a chemiluminescent and electrochemical signal [24,25,26]. By integrating such a dual detection method, the biosensor facilitates rapid CA IX detection, significantly reducing assay time compared to conventional ELISA while still maintaining a reasonable balance of sensitivity and specificity. Moreover, the herein proposed dual-mode approach reduces the risk of false readouts, showing improvement over the currently developed single-mode CA IX biosensors. To our best knowledge, this is the first time that both optical and electrochemical detection techniques have been systematically evaluated for ITO-based CA IX sensing, offering a more efficient and accessible platform for biomarker detection in cancer diagnostics.

## 2. Materials and Methods

The electrochemical sensors consisted of a glass slide with a patterned ITO electrode structure with a PDMS o-ring to keep an equivalent deposited liquid volume and area for all samples. Each step of material preparation is described below.

### 2.1. PDMS O-Ring Preparation

To fabricate o-rings, a stereolithography (SLA) resin 3D printer (Form 3+, Formlabs, Somerville, MA, USA) was used to produce a polymer resin mold (Grey V4 photopolymer, Formlabs, Somerville, MA, USA). The mold contained several channel-linked o-rings of 9.56 mm inner diameter and 3 mm height. This negative mold was employed to create positive polydimethylsiloxane (PDMS, Sylgard™ 184 Silicone Elastomer, Midland, MI, USA) o-rings. The PDMS components were mixed at a crosslinker-to-base ratio of 1:10 (*w*/*w*) in a planetary mixer for 1 min at 500 revolutions per minute (RPM). The resulting mixture was poured into the 3D-printed mold and degassed in a vacuum chamber to eliminate air bubbles. A polyethylene terephthalate (PET) sheet was placed over the mold, which was then secured in a custom-made holder and cured at 60 °C for 3 h. After curing, the silicone o-rings were extracted, the excess parts (connection channels) were cut off, and the o-rings were rinsed with 99.8% 2-propanol, followed by drying under a nitrogen stream. 

### 2.2. ITO Surface Preparation

ITO glass sheets (50 mm × 50 mm × 0.4 mm, 80 Ω) were sonicated in an ultrasound bath (10 min, at 30 °C, frequency 37 kHz, power 70%) in a 2% detergent solution (Hellmanex III, Hellma Analytics, Müllheim, Germany). Then samples were rinsed three times with deionized water (DIW), and the washing in an ultrasonic bath was repeated with the same parameters in DIW, acetone, and isopropanol. After the washing process, the ITO glass sheets were blow-dried under a nitrogen flow and treated by O_2_ plasma (O_2_ 1000 sccm, 800 W, 7 min) in a plasma chamber (GIGAbatch 360M, PVATePla, Feldkirchen (Munich, Germany)). After plasma treatment, the samples were manually cut with a diamond scriber (RV-129, UniTemp GmbH, Pfaffenhofen, Germany) into 1 cm × 1 cm pieces, and the particles were removed under nitrogen flow.

### 2.3. ITO Electrode Preparation

**Figure 1:**
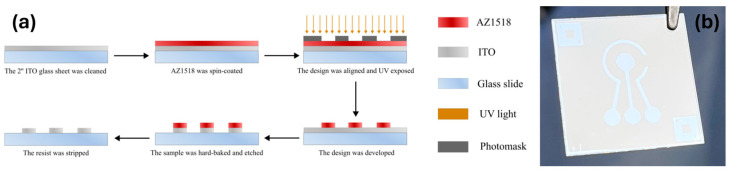
The indium tin oxide (ITO) electrode preparation process; (**a**) A schematic representation of the ITO sample fabrication; (**b**) An image of the fabricated ITO electrode.

A standard photolithography process shown in Figure 1a was utilized to fabricate the ITO electrodes. Prepared ITO glass sheets (25 mm × 25 mm × 0.4 mm, 80 Ω) were spin-coated (40 s, 4000 RPM, 4000 RPM/s acceleration) with a positive photoresist AZ1518 (MicroChemicals, Ulm, Germany). The photoresist was soft-baked on a hot plate at 100 °C for 2 min. The samples were then exposed via an iron oxide photomask in a mask aligner (Suss, MA6/BA6 Gen 4, Garching, Germany) utilizing an ND33 filter to achieve the final exposure dose of 70 mJ/cm^2^. After exposure, the samples were developed for 70 s in AZ 726 MIF developer (Microchemical, Ulm, Germany), rinsed with DIW, and blow-dried under nitrogen flow. Each structured slide was then hard-baked at 150 °C for 5 min then allowed to cool until room temperature. The ITO layer was etched in concentrated hydrochloric acid (35–38%) for 3–5 min, revealing the electrode pattern. The etching quality was evaluated by measuring the resistance of the etched parts with a multimeter. The photoresist layer was further removed by soaking the sample in acetone for 10 min while agitating the beaker. The prepared electrodes were then rinsed with isopropanol and blow-dried with compressed nitrogen. Finally, the samples were UV/ozone-treated in a UV/ozone cleaner (Novascan, Ames, IA, USA) for 20 min. The 3-electrode ITO device with a central working electrode (WE) (area 9.62 mm^2^), reference (RE), and counter electrodes (CE) is shown in Figure 1b. The coefficient of variation for the sensor’s design was 1.1% ± 1.7%, based on values from multiple measurements.

### 2.4. ITO Electrode Functionalization

**Figure 2:**
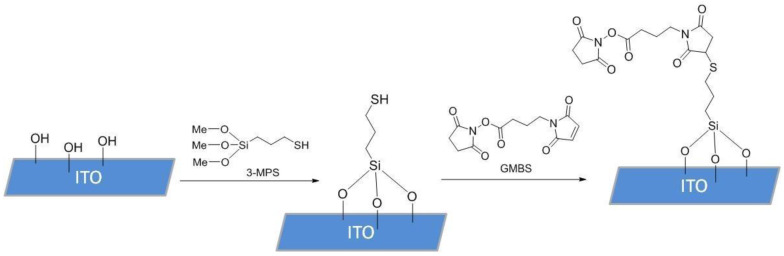
Functionalization scheme for the SAM deposition on the ITO surface. The first step included immobilization of 3-MPS onto the ITO surface containing -OH groups, which formed a SAM, whilst in the second step GMBS was applied, introducing an activated ester to ensure covalent binding of antibodies.

To functionalize the surface of the ITO samples, 10 µL of (3-mercaptopropyl) trimethoxysilane (3-MPS) 4% solution in methanol (MeOH, HPLC grade, 99.8%) was pipetted onto the surface of the electrode and incubated in a humidity chamber for 15 min. After incubation, the 3-MPS solution was removed from the sample with a micropipette, and the sample was rinsed 3 times with 100 µL absolute MeOH. A total of 10 µL of N-succinimidyl 4-maleimidobutyrate (GMBS) solution in MeOH (HPLC grade, 99.8%), prepared from 50 mg of GMBS in 0.5 mL dimethyl sulfoxide (DMSO) and diluted by 9.5 mL MeOH, were then introduced and incubated for 25 min. After incubation, the GMBS solution was removed, and the samples were washed 3 times with 100 µL of phosphate-buffered saline (PBS), containing 137 mM NaCl, 2.7 mM KCl, and 4.3 mM Na_2_HPO_4_. SAM deposition process is shown in Figure 2. The same process was also repeated with 3-MPS ethanol (EtOH, 99.8%) samples, where the incubation environment and solutions were prepared using absolute EtOH.

### 2.5. Sandwich Immunoassay Application to the Sample Surface

To functionalize the samples for the determination of limit of detection (LOD) and specificity tests for CAIX, an antibody pair with a capture (CAb) and unconjugated detection antibody (DAb) (ab253844, Abcam Limited, Cambridge, UK) was used. Prior to detection, DAb was conjugated to horseradish peroxidase (HRP) using a commercially available HRP conjugation kit (ab102890, Abcam Limited, Cambridge, UK) according to the manufacturer’s instructions. CA IX, CA II, and CA XII were produced and purified as described previously [27,28,29].

To assess protein binding efficiency for the functionalized ITO samples, initial tests for HRP attachment were performed. A PDMS ring was placed on the SAM-modified ITO glass surface, sealing the desired area. A total of 2 mg/mL of HRP solution was prepared in PBS, and 50 μL were deposited in the inner part of the PDMS ring. The solution was then incubated on the ITO sample surface for 30 min. After removing the applied solution with a pipette, the area was rinsed several times with DIW. Further, 50 μL of substrate mix from the ECL substrate kit (ab65623, Abcam Limited, Cambridge, UK) were placed on the ITO glass sheet, and the photoluminescence reaction intensity was measured spectrophotometrically.

**Figure 3:**
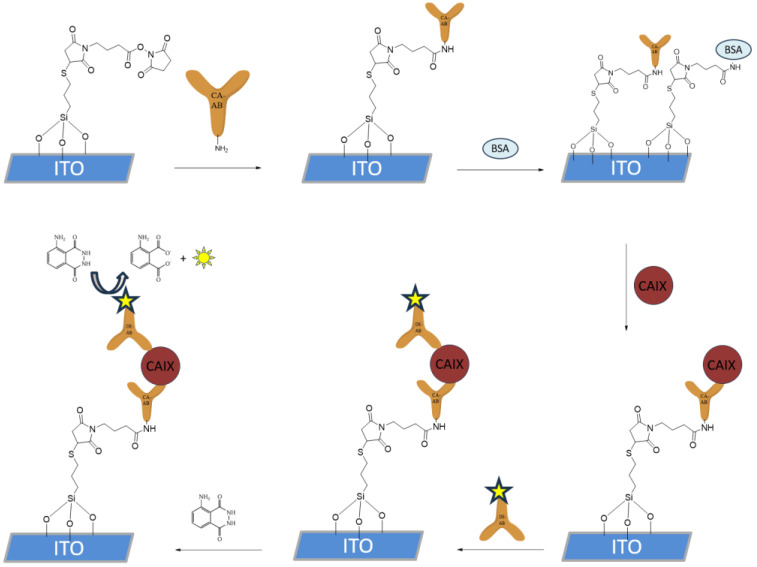
CAIX sandwich immunoassay immobilization on the silane SAM-functionalized ITO surface. Firstly, the capture antibody was introduced, after which the surface blocking with BSA was performed. HRP-spiked detection antibody was then introduced, after which the HRP-catalyzed oxidation of luminol was performed and detected with spectrophotometry and cyclic voltammetry.

The functionalization of the silanized ITO WE shown in Figure 3, with a surface area of 9.62 mm^2^, was performed by incubating 5 μL of CAb (1 μg/mL) solution in PBS for 30 min at room temperature in a humidity chamber. The electrodes were then thoroughly rinsed with PBS. To block the unreacted silane chemistry-based SAM, the electrodes were incubated in 10 μL of 1% bovine serum albumin (BSA) solution containing 0.1% Tween-20 in PBS for 30 min at room temperature in a humidity chamber. The electrodes were then rinsed and incubated with 5 μL of CA IX in PBS at the desired concentrations for the LOD samples, using PBS for the negative control samples, while for the specificity test, CA II or CA XII were added in a concentration of 10^−5^ g/mL. The incubation was performed at room temperature in a humidity chamber for 30 min. After rinsing, 5 μL of HRP-conjugated DAbs in PBS (1 μg/mL) were applied to the electrode surface and incubated for 30 min at room temperature. The electrodes were then rinsed with PBS and stored in PBS at 4 °C until further use.

### 2.6. The General Measurement Procedure

The electrochemical measurements were performed using a potentiostat/galvanostat (Autolab, Metrohm, Herisau, Switzerland) and a custom-made jig unless stated otherwise. The sample luminescence intensity was measured over the wavelength range of 300–800 nm using a custom-built measurement system. It consisted of an aluminum breadboard (MB2530/M, Thorlabs, Newton, NJ, USA), a photomultiplier tube (H8259-01, Hamamatsu Photonics, Shimokanzo, Iwata City, Shizuoka Pref., Japan), a complimentary photon counting unit (C8855-01, Hamamatsu Photonics, Shimokanzo, Iwata City, Shizuoka Pref., Japan), neutral density optical filters (NE10B and NE30B, Thorlabs, Newton, NJ, USA), optical filter holders (CFS1/M and CFS1-F1, Thorlabs, Newton, NJ, USA), a blacked-out container, a 3D-printed sample holder, as well as various optical element connectors. The optical signal was registered using the LabVIEW program (Austin, TX, USA) provided with the photon counting unit C8855-01 using a 20 ms sampling rate and a 10,000-point measurement. The measured spectral data was then processed and illustrated using the program Origin Pro 2024. To ensure equal liquid volume and even electrode coverage, in all experiments the PDMS o-rings were UV/ozone treated for 5 min, placed, and secured around the electrodes before proceeding further with the experiments. For both—electrochemical and optical measurements, a substrate mix from the enhanced chemiluminescence (ECL) substrate kit was applied to the ITO sample surface, which consisted of a luminol/reaction enhancer reagent (A) and a peroxide chemiluminescent detection reagent (B). For optical measurements, an ultra-high sensitivity ECL kit (ab133409, Abcam) was used to provide a higher luminescent signal, whilst for the electrochemical measurements, a standard ECL kit was utilized (ab65623, Abcam). In all cases, the substrate kit was mixed in a 1:1 ratio (*v*:*v*).

### 2.7. Electrochemical Measurements of the Samples

The electroactive surface area of ITO was characterized by utilizing cyclic voltammetry (CV) by using a 0.005 M potassium ferro-/ferricyanide redox system with 0.1 M KCl as the supporting electrolyte. Three scans with voltage sweeps from −1.1 V to 1.0 V vs. RE were performed by performing 9 consecutive measurements with varying scan rates of 10 mV/s to 500 mV/s. To characterize the electrode modification steps, CV measurements were carried out using a 0.005 M potassium ferro-/ferricyanide redox system with 1 M KCl in pH 7.4 PBS from −1.1 V to 1.0 V vs. RE with a scan rate of 100 mV/s. Additionally, surface wettability characterization was performed for the linker modification steps using contact angle measurements (Drop Shape Analyzer DSA25, A. Krüss Optronic GmbH, Hamburg, Germany) via measuring the angle of 10 µL drop of DIW with the modified ITO surface.

The specificity and LOD measurements were performed by depositing 100 μL of substrate mix from the standard ECL substrate kit onto the electrode before placing it into the custom-made jig. The experiments were performed by a CV sweep with 2 scans from −0.2 V to 0.7 V vs. RE with a scan rate of 50 mV/s, using only the peak cathodic potential of the first scan for the analysis.

## 3. Results and Discussion

### 3.1. Electrochemical Characterization of the ITO Biosensor

The electrochemical behavior of the ITO samples was characterized using CV with a 5.0 mM ferro/ferricyanide redox pair containing 0.1 M KCl at different scan rates from 10 mV/s to 500 mV/s as previously outlined in [30]. The electroactive surface area was then evaluated using the Randles-Sevcik equation:

I_p_ = (2.69 × 10^5^) × n^3/2^ × A × C × D^1/2^ × v^1/2^, (1)

where A is the electrode surface area (cm^2^), D is the diffusion coefficient (cm^2^/s), C is the concentration of the electroactive species in the bulk solution (mol/cm^3^), and v is the scan rate (V/s).

**Figure 4:**
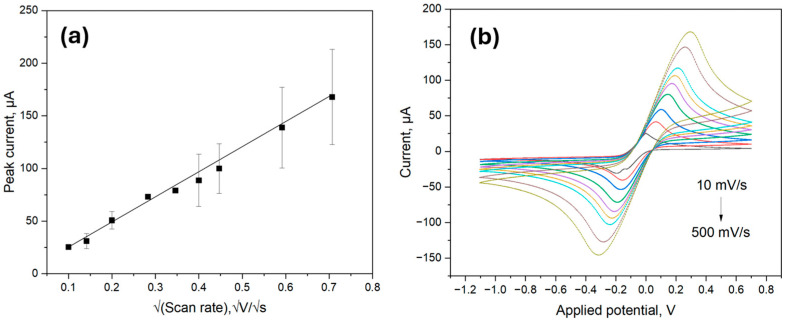
Electroactive surface area detection using the Randles-Sevcik method; (**a**) Linear relations of untreated ITO with the cathodic potential against the square root of scan rate; (**b**) The CV of untreated ITO electrode at different scan rates ranging from 10 to 500 mVs^−1^.

As shown in Figure 4a, the peak current values obtained from the CV scans visualized in Figure 4b were proportional to the square root of the scan rate; therefore, the electroactive surface area could be calculated by inserting the values of the constants in the Equation (1). The obtained electroactive surface area was thus 6.66 mm^2^, which was comparable to the calculated area of 9.62 mm^2^. The value mismatch could be attributed to measurement errors and the difference between electrically active surface area and total surface area.

**Figure 5:**
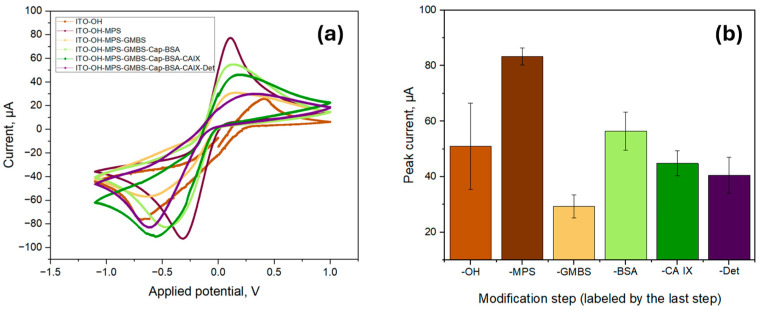
Surface characterization via cyclic voltammetry for the proposed biosensor; (**a**) Cyclic voltammograms for each of the functionalization steps; (**b**) The average peak current values of each functionalization step; the labels indicate the last deposition step prior to the measurement.

To further characterize the biosensor, the functionalization process was investigated by using CV measurements by using a 5.0 mM ferro/ferricyanide redox pair containing 1.0 M KCl in a pH 7.4 PBS solution as seen in Figure 5a. All measurements were made in triplicate, and the anodic peak current was averaged as per Figure 5b. The initial peak current value of the ITO surface (labelled as -OH) was found to be 5.10 × 10^−5^ A ± 1.55 × 10^−5^ A, which was increased to 8.32 × 10^−5^ A ± 0.31 × 10^−5^ A after the surface modification with a silane-based linker (MPS). The increase could be attributed to the formation of MPS SAM, which contains thiol groups with the ability to attract metal ions, thus increasing the conductivity of the surface [31]. The deposition of MPS onto the sensor surface considerably reduced its wettability, increasing the contact angle to 43.21° ± 2.96°, confirming the successful formation of a SAM. In contrast, the oxidized surface had extremely high wettability, making it impossible to measure the contact angle.

The deposition of GMBS onto the functionalized surface, however, led to a decrease in peak current to 2.93 × 10^−5^ A ± 0.42 × 10^−5^ A. The introduction of GMBS did not considerably alter the wettability of the surface as the measured contact angle remained nearly unchanged at 42.51° ± 1.92°, indicating that the surface hydrophobicity remained nearly unchanged. As GMBS contains a maleimide and succinimidyl ester moiety that react with the thiol moieties of MPS, the peak current decrease is likely attributed to the formation of a denser, more insulating layer. This layer hinders the diffusion of redox species to the electrode surface, thereby limiting electron transfer and resulting in a decrease in peak current. The peak current again increased to 5.64 × 10^−5^ A ± 0.68 × 10^−5^ A after the immobilization of CAb (indicated as -Cap) and surface blocking with BSA. This is likely due to the change in electron transfer kinetics of redox probes, influenced by the adsorption of BSA onto the surface of the electrode and the enhanced electron transfer caused by the immobilization of the capture antibody [32]. The peak current decreased after introducing CA IX and DAb (indicated as -Det) to 4.48 × 10^−5^ A ± 0.45 × 10^−5^ A and 4.05 × 10^−5^ A ± 0.65 × 10^−5^ A, respectively, due to specific interactions of CA IX and DAb within the previously deposited biological stack.

### 3.2. Proof-of-Concept Optical Measurements

To assess protein binding on the ITO samples, the comparison between samples functionalized with linker molecules and those without such functionalization was performed. All samples were incubated with HRP solution to evaluate binding efficiency. For sample functionalization, two approaches were tested. The first approach used dry ethanol as a solvent for attaching 3-MPS and GMBS, as detailed in the methods section. The second approach used methanol as a solvent. While the published data [33] emphasized the need for dry ethanol, experiments were performed in a laboratory setting, thus raising concerns about the humidity; subsequently, dry methanol was utilized in the experiments. A PDMS o-ring with an inner area of 71.86 mm^2^ was placed on the ITO glass, sealing and securing a constant area and avoiding any fluid leakage during functionalization of the ITO surface, electrochemical, and optical measurements. A mix of ECL substrate kit (Ultra High Sensitivity (ab133409), Abcam) was used for these measurements.

**Figure 6:**
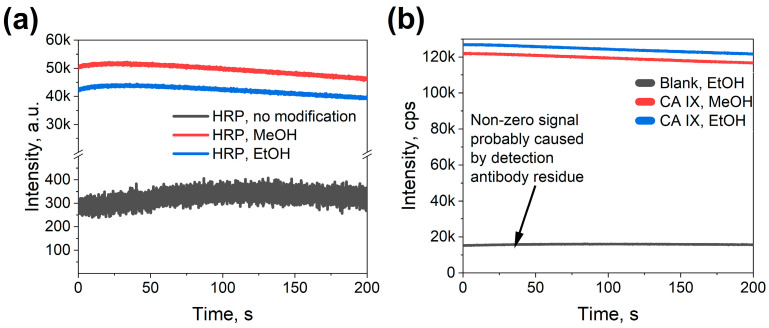
Proof-of-concept optical measurements on a 71.86 mm^2^ large ITO surface; (**a**) Signal comparison for linker-HRP samples using methanol or ethanol as solvents with a linker-free sample as the control; (**b**) Signal comparison for linker-CA IX stack samples using methanol or ethanol as solvents.

As shown in Figure 6a, both ethanol- and methanol-treated ITO samples, where these solvents were used for the linker deposition, registered a higher luminescence intensity as compared to the unfunctionalized linker-free samples. This shows that both approaches to functionalization were capable of increasing protein binding to the ITO surface at least 10^2^-fold, with minimal nonspecific binding observed.

After confirming the successful binding of HRP protein molecules to the functionalized ITO surfaces, the next step was to test ITO performance in the CA IX immunoassay. For that, several ITO samples were prepared and consecutively coated in protein solutions to build the protein stack on the surface. The samples were then measured for their luminescence intensity. As highlighted in Figure 6b, the assembly of the stack was successful; however, the background intensity of ITO samples was significantly higher when compared to the HRP samples (signal-to-noise ration\ ~7 instead of ~10^2^). This subsequently could be attributed to the incomplete removal of detection antibodies during rinsing. Having established the successful functionalization of ITO surfaces, the next step was to evaluate the biosensor’s specificity by assessing its ability to distinguish CA IX from structurally similar carbonic anhydrases.

### 3.3. The Determination of the Sensor Specificity

**Figure 7:**
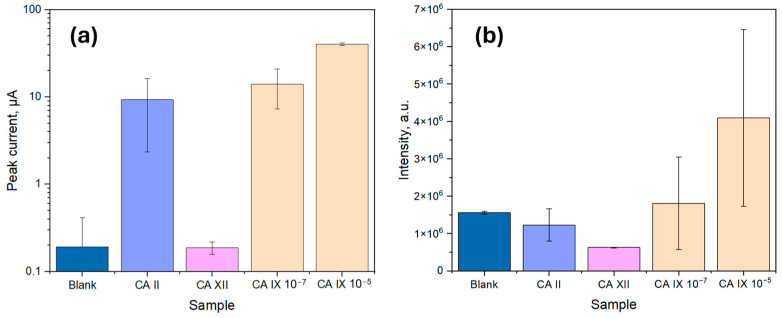
Biosensor specificity results with CA II and CA XII at a concentration of 10^−5^ g/mL compared to a blank stack and CA IX with two concentrations (10^−7^ g/mL and 10^−5^ g/mL); (**a**) Based on the peak current values from cyclic voltammetry; (**b**) Based on the integrated area under the intensity curve in spectrophotometry.

To evaluate the specificity of the sandwich immunoassay, two additional carbonic anhydrase isoforms, CA II and CA XII, were tested and compared to varying concentrations of CA IX, as shown in Figure 7. Both CA II and CA XII are structurally related to CA IX and are present in trace amounts in human serum and urine [12]. Due to their structural similarities, assessing the immunoassay’s specificity against these isoforms is crucial to minimize cross-reactivity and prevent false-positive results [34].

Based on the results in Figure 7, as expected, the blank sample exhibited the lowest signal, as it lacked CA IX molecules, preventing CA capture and resulting in minimal signal for the electrochemical detection method (1.93 × 10^−7^ A ± 2.22 × 10^−7^ A), while for the optical detection method the signal was considerable (1.6 × 10^6^ a.u. ± 0.4 × 10^6^ a.u.). The observed background can be attributed to the slight non-specific adsorption of HRP-conjugated DAb, which catalyzed the luminol reaction in low amounts, creating a background signal for the optical method. Electrochemistry, on the other hand, is known to facilitate the luminol reaction due to the oxidation of luminol when a positive potential is applied, producing a background signal [35]. Regarding the sensor specificity, CA XII showed a signal intensity comparable to or lower than the blank value for both methods, confirming the high specificity of the biosensor for CA IX over CA XII. However, CA II exhibited a signal comparable to that of CA IX at 100 ng/mL, likely due to structural similarities among the carbonic anhydrases, leading to expected non-specific binding for both sensors.

For optical measurements, the signal intensity for CA IX at 10^−7^ g/mL exhibited high variability (1.8 × 10^6^ a.u. ± 1.2 × 10^6^ a.u.), with some overlap between the non-specific binding response of CA II (1.2 × 10^6^ a.u. ± 0.4 × 10^6^ a.u.) and the blank value (1.6 × 10^6^ a.u. ± 0.4 × 10^6^ a.u.). However, at 10^−^^5^ g/mL, the average CA IX signal was distinctly higher (4.1 × 10^6^ a.u. ± 2.3 × 10^6^ a.u.), indicating a detection threshold within this range. Despite this, the possibility of false positives in optical detection cannot be excluded, particularly due to background signal contributions from non-specific interactions. The observed variability in optical measurements suggests that in real-world applications, matrix effects, cross-reactivity, or other artifacts may contribute to signal fluctuations, emphasizing the need for further optimization regarding surface blocking, fouling, and substrate reagent choice.

In contrast, electrochemical measurements showed a peak current increase significantly above the background signal (1.93 × 10^−7^ A ± 2.22 × 10^−7^ A) at a CA IX concentration of 10^−^^7^ g/mL (1.41 × 10^−5^ A ± 0.68 × 10^−5^ A), showing a further rise at 10^−^^5^ g/mL (4.01 × 10^−5^ A ± 0.13 × 10^−5^ A). CA II, however, displayed a comparable response (0.93 × 10^−5^ A ± 0.70 × 10^−5^ A) at lower concentrations. Despite this, the electrochemical method demonstrated reduced variability, resulting in more consistent peak signals for CA IX detection. The enhanced electrochemical response is likely due to the combined effects of the luminol oxidation reaction, which is facilitated by the applied potential and the catalytic properties of the immobilized biological stack. Given the lower signal fluctuations and improved reproducibility, the electrochemical detection method was selected for further analysis.

### 3.4. Electrochemical Measurements of CA IX Detection Limit

**Figure 8:**
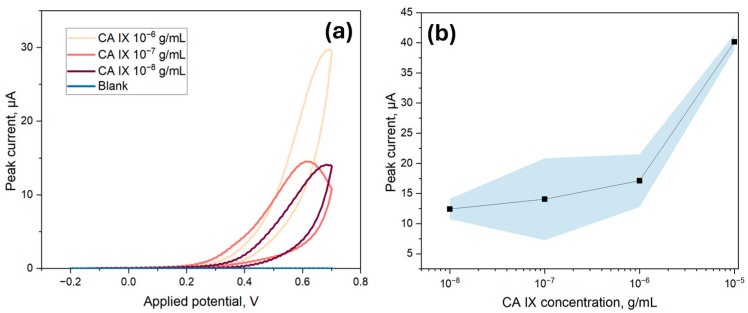
LOD analysis for cyclic voltammetry measurements of the CA IX sandwich immunoassay with different concentrations of CA IX (10^−5^ g/mL to 10^−8^ g/mL); (**a**) Examples of the CV curves obtained in the measurements where the peak current value is visible around 0.6 V; (**b**) The herein obtained LOD curve with the area in blue showing a 95% confidence interval. The LOD, calculated from 3.3 times the standard deviation of the blank divided by the slope of the calibration curve, was 266.4 ng/mL.

To determine the LOD of the ITO-based biosensor, various concentrations of CA IX, ranging from 10 ng/mL to 10 µg/mL, were immobilized onto the surface of the biosensor. For these experiments, the ECL substrate kit was utilized, which had a cathodic potential peak at 0.6 V for electrochemical detection. Thus, a CV range from −0.2 V to 0.7 V was utilized to capture the peak signal. As indicated in Figure 8a, the cathodic peak signal at 0.6 V from the second scan was used for the LOD analysis, as it provided a clearer and more stable signal excluding surface-related artifacts. Although the electrodes were tested under the same conditions, slight shifts in the applied potential corresponding to the peak current could be observed, likely due to minor variations in electron transfer kinetics or local diffusion effects. The peak current values were then averaged and plotted in respect to the corresponding concentrations for the calibration curve. As per Figure 8b, a linear correlation was found between the immunosensor response and the concentration of CA IX with an equation of I_p_ (A) = 1.27 × 10^−5^ + 2.75 × [CA IX] (R^2^ = 0.998), from which slope and intercept error were calculated to be 0.20 and 1.541 × 10^−6^, respectively. The LOD for the electrochemical method was calculated to be 266.4 ng/mL, which was calculated as 3.3 times the standard deviation of the blank divided by the slope of the calibration curve. Additionally, as indicated in Figure 8a, the blank value was found to be very low, further contributing to the high sensitivity of the detection method. The limit of quantification was 807.3 ng/mL, taken as ten times the standard deviation of the blank divided by the slope of the calibration curve.

While the obtained LOD of 266.4 ng/mL falls within the nanogram-per-milliliter range, it remains higher compared to conventional ELISA-based assays, which have demonstrated LODs as low as 2.5 pg/mL [36]. Commercial ELISA kits, such as those provided by R&D Systems, typically offer detection ranges from approximately 16 pg/mL to 1000 pg/mL. However, ELISA-based methods generally require several hours to complete, along with specialized laboratory equipment and trained personnel, making them less suitable for rapid or point-of-care applications. Similarly, immunoprecipitation-based sensors have achieved an LOD of around 0.1 ng/mL [37], yet their extensive sample preparation procedures and labor-intensive workflows limit their practical feasibility in routine clinical settings. Although the proposed biosensor does not currently surpass these methods in terms of absolute sensitivity, it offers significant advantages, including faster detection times, simpler operational protocols, and greater potential for point-of-care implementation.

Nevertheless, considering previously reported urinary CA IX concentrations ranging from 20 pg/mL to 3 ng/mL in patients with renal cell carcinoma, with control samples containing 0 to 2 pg/mL [12], the naturally occurring urinary CA IX levels remain several orders of magnitude lower than the herein presented assay’s LOD and LOQ. Given that the detection threshold is in the ng/mL range, it is unlikely that this assay, in its current form, would be suitable for direct detection of CA IX in human urine without prior sample concentration or enrichment. Subsequently, a potential approach to improving detection capability is to enhance assay sensitivity by optimizing antibody affinities, optimizing the background signal by using a different reaction substrate for the electrochemical detection, or using signal amplification strategies, for instance, by increasing the surface area of the working electrode [38,39]. Additionally, a dual-modality approach combining both of the herein proposed methods could further enhance the detection sensitivity of CA IX by providing an internal result control, reducing the risk of false negatives and positives. Thus, further work should be focused on further optimizing both the electrochemical and optical components of the system to enhance the detection sensitivity.

Further consideration for real-world applications should also address biosensor stability, shelf-life, and environmental influences that may affect long-term performance. Additionally, the lack of evaluation in biological matrices, such as serum or urine, represents a limitation, as potential matrix effects, biofouling, and nonspecific adsorption could impact assay reliability. Future works should therefore focus not only on optimizing the electrochemical and optical components of the system to enhance sensitivity but also on assessing the sensors robustness in complex biological environments to ensure feasibility beyond controlled laboratory conditions. By systematically addressing these challenges and optimizing the system, the herein proposed CA IX biosensing platform can be further advanced into a practical and reliable diagnostic tool.

## 4. Conclusions

In conclusion, this study presents a novel approach for the functionalization of ITO surfaces to enable dual-mode detection of the hypoxia-associated biomarker CA IX. By employing silane-based self-assembled monolayers, we successfully immobilized CA IX-specific antibodies, facilitating selective target capture. The biosensor was characterized using both optical and electrochemical detection. The specificity of the sensor against CA XII, an isoform of CA IX, showed to be good for both sensing methods, while the specificity against CA II was reduced due to similarities between the isoforms. Nevertheless, selectivity tests against CA II and CA XII confirmed that electrochemical detection exhibited higher specificity and reproducibility, whereas optical detection showed higher variability due to non-specific interactions and background signal contributions. The electrochemical measurements provided a more reliable and consistent signal, likely due to the symbiotic relationship of the enhanced luminol oxidation in the presence of both HRP and applied potential, yielding a limit of detection of 266.4 ng/mL.

This work highlights the potential of ITO-based biosensors in biomedical applications, particularly in non-invasive cancer biomarker detection, due to the integration of optical and electrochemical modalities offering a complementary approach that enhances measurement reliability. Future efforts should focus on further optimizing detection sensitivity and exploring strategies for improving specificity in optical readouts. Overall, this study contributes to the development of advanced biosensing platforms and expands the applicability of ITO in dual-mode detection systems for clinical diagnostics.

## Data Availability

Dataset available on request from the authors.

## Abbreviations

The following abbreviations are used in this manuscript:

ITO	Indium tin oxide
CA	Carbonic anhydrase
HRP	Horseradish peroxidase
LOD	Limit of detection
SAM	Self-assembled monolayer
ELISA	Enzyme-linked immunosorbent assay
SLA	Stereolithography
PDMS	Polydimethylsiloxane
PET	Polyethylene terephthalate
DIW	Deionized water
MeOH	Methanol
EtOH	Ethanol
3-MPS	(3-mercaptopropyl) trimethoxysilane
GMBS	N-succinimidyl 4-maleimidobutyrate
DMSO	Dimethyl sulfoxide
PBS	Phosphate-buffered saline
CAb	Capture antibody
DAb	Detection antibody
BSA	Bovine serum albumin
ECL	Enhanced chemiluminescence
RE	Reference electrode
CE	Counter electrode
WE	Working electrode
CV	Cyclic voltammetry
RPM	Revolutions per minute
